# Natural Products: Advances in Isolation, Characterization, Biological Activities, and Plant Protection Applications

**DOI:** 10.3390/molecules31162921

**Published:** 2026-08-21

**Authors:** Hazem S. Elshafie

**Affiliations:** Department of Agricultural, Forestry, Food and Environmental Sciences (DAFE), University of Basilicata, Via dell’Ateneo Lucano 10, 85100 Potenza, Italy; hazem.elshafie@unibas.it; Tel.: +39-0971-205498; Fax: +39-0971-205503

## Abstract

Natural products derived from plants and microorganisms remain promising alternatives to synthetic chemicals and conventional substances across a wide range of fields, including pharmaceuticals, agriculture, and industry. They also play an important role in the development of sustainable biopesticides and biofertilizers. Chemically, natural products can be grouped into overlapping structural and biosynthetic categories, including alkaloids, terpenoids, flavonoids, glycosides, saponins, and polyketides. Each group reflects distinct chemical characteristics and associated biological functions. This overview highlights the main findings from the *Molecules* Special Issue: “Natural Products: Isolation, Analysis and Biological Activity, 2nd Edition”, focusing on three categories: (i) plant protection, plant health, and plant biotechnology; (ii) functional foods and health-promoting formulations; and (iii) therapeutic natural products and derivatives. Exploring the mechanisms of action of natural products is essential for ensuring safety and optimizing sustainable resource use. This understanding drives critical innovations across drug discovery and materials science. Ultimately, such research reinforces their cross-disciplinary significance for advancing human health and promoting environmental sustainability.

## 1. Introduction

Plant and microbial natural products are essential sources of bioactive compounds that significantly impact medicine, agriculture, and industry [1,2]. These include various phytochemicals and microbial metabolites with unique biochemical properties contributing to their therapeutic efficacy. Many modern pharmaceuticals originated from these natural sources. The development of aspirin was historically inspired by naturally occurring salicylates from willow, whereas penicillin was discovered as a fungal metabolite. Beyond their medicinal applications, these compounds also play crucial roles in ecological interactions [3,4], aiding plant defence mechanisms [5] and microbial communication [6]. As the demand for natural alternatives to synthetic chemicals grows, the exploration of these products is vital for advancing health and sustainability.

In agriculture, natural products are increasingly recognized for their role in sustainable practices, serving as biopesticides and biofertilizers that enhance soil health, promote plant growth, and reduce reliance on synthetic compounds [7]. Plant essential oils (EOs) can effectively combat pests and pathogens, playing a vital role in integrated pest management strategies essential for ensuring food security [8,9]. Additionally, microbial metabolites, especially those derived from fungi and bacteria, can help discover novel antibiotics and bioactive compounds to combat antibiotic resistance effectively [10]. Studying natural products deepens our understanding of nature’s biochemical potential, fostering new technologies and therapies that improve human health and well-being and promote environmental sustainability.

## 2. Main Groups of Natural Products

Natural products are diverse organic compounds produced by plants, animals, and microorganisms. They are generally classified into two groups: primary metabolites and secondary metabolites. Primary metabolites are essential molecules (carbohydrates, lipids, amino acids, and nucleic acids) that sustain life through basic functions like energy production, growth, and nutrient use [1]. In contrast, secondary metabolites comprise specialized compounds with diverse ecological and biological functions, including alkaloids involved in defence, terpenoids in signalling and protection, and phenolic compounds in antioxidant and defensive responses [6]. Importantly, these metabolites can be classified into overlapping structural and biosynthetic categories, as different compounds may share common structural features and biosynthetic origins. These compounds exhibit a wide range of biological activities and play important roles in plant defence, ecological interactions, pharmacology, agriculture, and biotechnology [2,7].

## 3. Summary of Contributions Published in the Special Issue

This Editorial provides an overview of the most significant papers published in Molecules within the Special Issue titled “Natural Products: Isolation, Analysis and Biological Activity, 2nd Edition”. The published research encompasses three main categories: (i) plant protection, plant health, and plant biotechnology (Contributions 1–3); (ii) functional foods and health-promoting formulations (Contributions 4 and 5); and (iii) therapeutic natural products and derivatives (Contributions 6–8).

### 3.1. Plant Protection, Plant Health, and Plant Biotechnology

The current Special Issue published three important papers related to applied biocontrol and agricultural protection. This category focuses on using natural substances (plant extracts, bacterial metabolites, and fertilizers) to protect crops from diseases and enhance growth.

The current Special Issue includes three important papers addressing biocontrol, plant tissue culture, and plant–nematode interactions. These studies highlight the potential of natural products and beneficial microorganisms for agricultural protection and tissue culture approaches, as well as the interactions between plants and nematodes and their implications for crop health. In particular, Horoszkiewicz et al. (Contribution 1) have evaluated plant extracts and bacterial supernatants for controlling wheat fungal diseases. Garlic extract was rich in allicin, betulin, and minerals, while Jerusalem artichoke (*Helianthus tuberosus*) showed higher phenolic content. The supernatants of Enterobacter and Paenibacillus displayed distinct metabolic profiles and showed strong antifungal activity in vitro and reduced disease severity in greenhouse trials. These results position *H. tuberosus* extract as a promising candidate for sustainable wheat protection programs.

The study conducted by Tudruj et al. (Contribution 2) examined the effects of L-tryptophan on flax callus cultures and found that its application at 1 mM effectively stimulated the accumulation of bioactive compounds, including carotenoids and polyphenols. L-Tryptophan also induced changes in cell wall composition by reducing cellulose and lignin levels and increasing pectin content. These findings highlight the potential of L-tryptophan as a modulator of secondary metabolism and cell wall dynamics in plant tissue cultures. The study of Dinh et al. (Contribution 3) has identified a new nematode species, *Scutellonema curcumae*, in Vietnamese turmeric (*Curcuma longa* L.) through morphological and molecular analyses. The study found an association between nematode population density, phytochemical composition, and bioactivity of the host plant. This first report highlights the potential links between nematode dynamics, yield-related traits, and medicinal quality, suggesting that nematode management may be an important consideration in precision agriculture.

### 3.2. Functional Foods and Health-Promoting Formulations

Functional foods and health-promoting formulations have attracted increasing attention because of their potential to improve health beyond basic nutrition. Natural products and plant-derived ingredients are being explored for their ability to prevent or manage chronic diseases and promote overall well-being. This Special Issue presents two significant papers highlighting recent advances in the development and evaluation of functional ingredients with potential health benefits. In particular, the study by Gościniak et al. (Contribution 4) optimized the extraction process of *Amelanchier alnifolia* leaves using Plackett–Burman and Box–Behnken designs, leading to improved recovery of bioactive compounds and enhanced phenolic content. Comparative analysis among different cultivars revealed notable variations in polyphenol composition. Furthermore, the incorporation of oligosaccharides into lyophilized systems helped preserve bioactive properties, improve chlorogenic acid release, and enhance prebiotic activity. FT-IR analysis confirmed the interactions between polyphenols and oligosaccharides. Overall, these systems have potential as functional ingredients for supporting diabetes management and gut health, with further in vivo studies required.

The study carried out by Unno et al. (Contribution 5) has evaluated the suppressive effects of green tea on the relatively early stress response in male mice using confrontation rearing as an experimental model. In particular, the stress-relieving potential of green tea has been evaluated by investigating the balance between caffeine (C), epigallocatechin gallate (E), theanine (T), and arginine (A), expressed as the CE/TA ratio. Researchers tested different brewing conditions and found that, using green tea infusion samples prepared under different conditions and model solutions containing C, E, T, and A, stress-reducing effects were associated with a CE/TA ratio below 2 and a sufficient theanine concentration. Tea infusions prepared under appropriate conditions achieved a lower CE/TA ratio and maintained the required theanine level, whereas tea consumed as a powder with a high CE/TA ratio did not show a stress-relieving effect. The results showed that the functional effect of green tea may depend on the relative proportion of antagonistic compounds (CE) to beneficial amino acids (TA), as well as the extraction conditions controlling their release into the infusion.

### 3.3. Therapeutic Natural Products and Derivatives

This study comprises one original research article and two comprehensive reviews, collectively examining the chemical synthesis, structural characterization, and pharmacological profiling of natural compounds for targeted therapeutic intervention in diseases such as cancer.

Tian et al. (Contribution 6) synthesized and characterized a series of new lupeol-3-carbamate derivatives and evaluated their biological activities. Most compounds showed improved anti-proliferative activity against cancer cell lines compared to lupeol. Salt formation enhanced both activity and solubility. Mechanistic studies indicated apoptosis induction via PI3K/AKT/mTOR pathway inhibition. This research provides a theoretical basis for designing potential anticancer leads.

In their review study (Contribution 7), Günther and Bednarczyk-Cwynar examine rare pristimerin dimers derived from Celastraceae plants, with particular emphasis on their structural diversity, linkage patterns, and isomeric forms. The authors discuss modern spectroscopic techniques employed for structural elucidation, as well as the biosynthetic pathways that involve quinonemethide reactivity. Compared with their monomeric counterparts, these dimers generally exhibit limited biological activity, which points toward possible alternative ecological roles. While the review identifies key directions for future research, it is important to note that the proposed ecological functions remain speculative and should be treated as working hypotheses pending rigorous experimental validation.

The review by Capasso et al. (Contribution 8) focused on epigallocatechin gallate (EGCG), the predominant catechin in green tea, known for its potent antioxidant, anti-inflammatory, and antitumor properties. Its gut microbiota-mediated metabolism enables systemic effects, modulating pathways linked to oxidative stress and lipid metabolism. EGCG has shown potential biological effects relevant to chronic diseases like cancer and cardiovascular disorders. However, its systemic and clinical effects remain incompletely understood, highlighting the need for further research to clarify its mechanisms, efficacy, safety, and potential therapeutic applications.

## 4. Conclusions

The study of natural products is crucial for advancing scientific knowledge, improving health outcomes, fostering sustainability, and preserving biodiversity. Their diverse applications underscore the need to integrate natural product research across multiple scientific disciplines. However, translating laboratory discoveries into practical use requires a rigorous approach: comprehensive chemical characterization, mechanistic studies to explore modes of action, safety assessments, production standardization, and quality control, as well as validation through controlled laboratory experiments, field studies, and preclinical animal models. These steps are essential for establishing efficacy, reproducibility, and safety. Particular emphasis on mechanism of action is essential for uncovering new therapeutic targets and guiding the rational design of improved derivatives.
